# Creutzfeldt–Jakob Disease: An Unusual Presentation of Corticobasal Syndrome

**DOI:** 10.7759/cureus.11393

**Published:** 2020-11-09

**Authors:** Grant P Gosden, Lela Okromelidze, Sukhwinder Johnny S Sandhu, Erik H Middlebrooks

**Affiliations:** 1 Radiology, Mayo Clinic, Jacksonville, USA

**Keywords:** creutzfeldt–jakob disease, corticobasal syndrome

## Abstract

Corticobasal syndrome is an atypical parkinsonian syndrome consisting of a constellation of clinical findings that can be the result of various etiologies. While most cases are a result of a tauopathy, such as corticobasal degeneration, other etiologies must be considered in the evaluation of patients presenting with corticobasal syndrome. We present a case of a patient presenting with clinical features of corticobasal syndrome due to a prion disease, Creutzfeldt-Jakob disease (CJD), who was initially misdiagnosed due to known pitfalls in the CJD diagnostic criteria. We further discuss this unusual manifestation of CJD presenting as corticobasal syndrome and relevant diagnostic consideration in the evaluation of this entity.

## Introduction

Parkinsonian syndromes represent an array of typical and atypical disorders commonly including Parkinson’s disease, progressive supranuclear palsy, multiple system atrophy, and corticobasal syndrome (CBS). Differentiating these disorders clinically can often be challenging. CBS, an atypical parkinsonian syndrome, often manifests with asymmetric parkinsonism, myoclonus, apraxia, and dystonia. CBS is often related to a tauopathy, such as corticobasal degeneration (CBD), but other etiologies can less commonly produce a similar presentation of CBS.

## Case presentation

**Figure 1:**
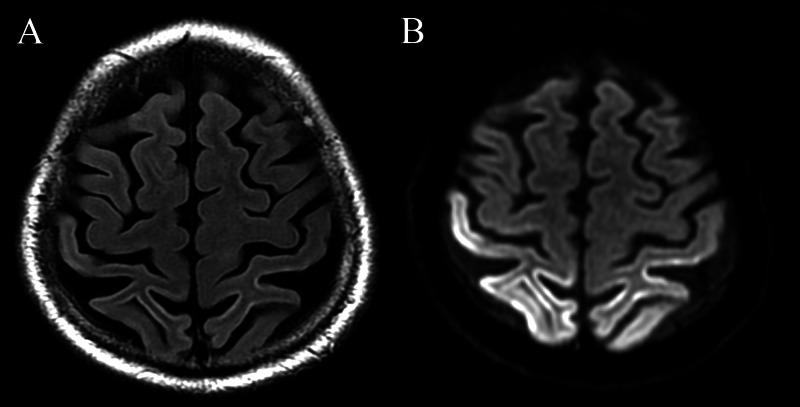
Outside MRI from one year prior to current presentation. (A) Axial FLAIR and (B) diffusion-weighted imaging show areas of diffusion restriction and corresponding FLAIR hyperintensity involving the bilateral peri-Rolandic cortex, greater in the right hemisphere. FLAIR, fluid-attenuated inversion recovery

A 68-year-old man presented with a three-year history of left arm “shaking” followed by progressive difficulty performing daily tasks with the left arm. He also endorsed trouble maintaining his balance. Select images from an MRI of the brain performed at an outside institution one year prior are shown in Figure 1.

At the time of MRI, cerebrospinal fluid (CSF) examination performed at an outside institution revealed two white blood cells with protein of 33 mg/dL and glucose of 66 mg/dL, and was negative for 14-3-3 protein (exact testing procedure was unknown). Cytological examination and serologic analysis for herpes simplex virus, syphilis, and Lyme disease were unremarkable. EEG revealed intermittent slowing in the left and right temporal lobes. Ioflupane SPECT (single-photon emission CT) scan was negative, and the patient was started on levodopa to no effect. The patient sought a second opinion one year later due to significant progression of symptoms.

Physical examination revealed low amplitude jerks in the left phalanges suggestive of myoclonus with evidence of dystonia primarily affecting the phalanges. There was also facial action myoclonus within the zygomaticus major and significant difficulty with mirroring of movements. Ideomotor apraxia was suspected. Tandem walking was notably impaired, even with guidance. The patient also struggled to remove shoes and socks and did not use his left arm. Surface EMG (electromyography) was also performed showing myoclonus that was most evident with action and stimulus sensitivity, which primarily affected the left upper extremity. There was no definite rest or postural myoclonus. Dystonia was also present, primarily affecting the left phalanges. Autonomic testing revealed no evidence of autonomic failure.

**Figure 2:**
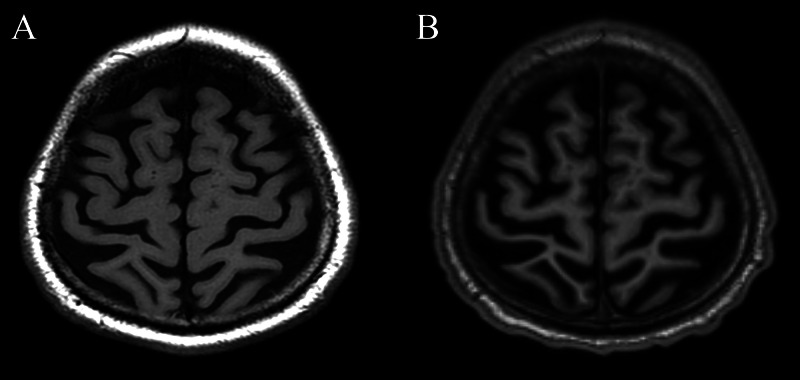
(A) Axial T1-weighted images from the initial MRI and (B) repeat MRI performed one year after initial MRI shows interval progression of peri-Rolandic atrophy (arrows), greater in the right hemisphere.

**Figure 3:**
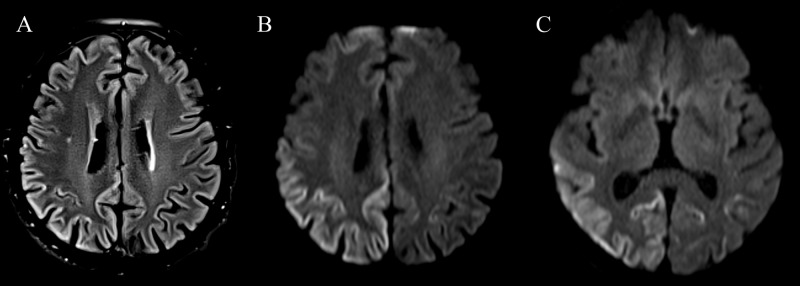
(A) Axial FLAIR and (B,C) axial diffusion-weighted images more inferiorly show persistent cortical FLAIR hyperintensity (arrow in A) and cortical diffusion restriction extending more inferiorly into the posterior temporal and occipital cortex (arrows in B and C). FLAIR, fluid-attenuated inversion recovery

Repeat MRI confirmed interim progression of peri-Rolandic atrophy (Figure 2) and persistent cortical diffusion restriction (Figure 3).

There was a clear progression of atrophic changes in a pattern that would fit with CBD; however, the pattern of cortical diffusion restriction was more concerning for underlying Creutzfeldt-Jakob disease (CJD) as a potential mimic. Repeat CSF examination was ordered to confirm the diagnosis of CJD. At this time, 14-3-3 protein was positive and a diagnosis of CJD was made.

## Discussion

CJD is the most common human prion disease. Like other prion diseases, CJD results from the conversion of normal brain protein into misfolded disease-causing forms. Previously limited to CSF analysis and brain biopsy, MRI has relatively recently been recognized as the third pillar of diagnosing the disease [1]. Studies have shown that proper MRI analysis has sensitivity and specificity for probable CJD of >90% [2]. Additional studies have also shown that MRI may be more accurate than CSF analysis for the diagnosis of CJD. In this patient, an overreliance on CSF markers prevented the correct diagnosis from being identified.

Diffusion restriction within the basal ganglia and/or thalamus is a commonly recognized radiographic pattern of CJD; however, more than one-third of cases may have isolated cortical imaging changes [3]. Only recently have the Centers for Disease Control and Prevention (CDC) MRI diagnostic criterion been updated to include diffusion restriction or FLAIR (fluid-attenuated inversion recovery) hyperintensity in the caudate/putamen or two or more cortical areas (temporal, occipital, or parietal) [4]. CJD also commonly spares the peri-Rolandic region, but it can occasionally have similar imaging features as in this case [3]. Cortical diffusion restriction has a broad differential diagnosis, such as seizure activity, infarction, and hyperammonemia. The absence of any supportive history or diagnostic findings of an alternative cause, as well as the progressive atrophy and absence of ictal events, makes these unlikely. 

The initial suspicion in this patient was CJD after the first MRI was performed; however, this diagnosis was excluded due to a negative 14-3-3 protein. Interestingly, false-negative results of 14-3-3 protein have been reported to occur in nearly half of patients with a CBS presentation of sporadic CJD [5]. Various lab tests are available for CJD diagnosis. The 14-3-3 protein detection test is one of the most common used for detecting 14-3-3 protein in CSF, but unfortunately test sensitivity and specificity vary in the range of 80-90% [6]. Furthermore, false-positive results have been observed in patients with various neurologic diseases, such as cerebral metastases, paraneoplastic diseases, and herpes simplex encephalitis [7]. Elevated tau protein in CSF is yet another marker looked for in CJD. Importantly, increase in non-phosphorylated tau levels in CSF is noted in these patients [8]. Real-time quaking-induced conversion (RT-QuIC) of CSF developed for CJD detection reported 91% sensitivity and 98% specificity [9], with RT-QuIC of nasal brushing also showing promising results [10]. Despite the variety of existing diagnostic tests, pathological studies of the brain remain the gold standard, with unfortunately many of them taking place postmortem [11]. While it is unknown which methods were initially performed at the outside hospital, repeat testing showed a positive CSF assay, positive RT-QuiC, and positive T-tau protein. 

The patient’s physical examination findings share features of CBS, which could lead to an erroneous clinical diagnosis. Patients presenting with CBS due to CJD commonly experience limb apraxia and alien limb phenomena starting on the non-dominant side in more than half of patients. Other frequent features include sensory loss, dysphasia, and neglect. Myoclonus, dystonia, and rigidity are commonly present, in addition to gait ataxia pyramidal dysfunction and cognitive impairment [5].

The patient also had a negative ioflupane SPECT scan and was unresponsive to levodopa, which does not support a diagnosis of Parkinson’s disease. With the rapid progression of symptoms and imaging findings, as well as lack of dysautonomia, multiple system atrophy is unlikely. The most conclusive evidence is the MRI findings, which initially suggested CJD. MRI findings can often times be missed in patients with CJD and are crucial to the diagnosis. These findings for CJD have a tendency to be overlooked, especially at institutions that may have less exposure to the disease. For those institutions that are less likely to encounter CJD, maintaining it as a differential is important to ensuring that it is not missed [12].

## Conclusions

While the case presented was an atypical presentation of CJD, it emphasizes the value of MRI in diagnosis. Maintaining a differential diagnosis of CJD in patients with CBS signs and symptoms remains important. In the initial presentation and after initial MRI, CJD was suspected. This diagnosis was not pursued due to negative 14-3-3 protein; however, the radiological findings are able to correctly distinguish the signs of CJD prior to the laboratory testing results. This case highlights the importance of comprehensive decision-making to integrate physical examination findings, radiological findings, and laboratory findings, as well as the need for longitudinal follow-up in cases where there is high suspicion for CJD but not initially meeting established diagnostic criteria.
